# Rifampicin for Treatment of Cholestatic Pruritus Caused by Drug-Induced Acute Liver Injury as Assessed by the RUCAM Classification

**DOI:** 10.1155/2020/8872804

**Published:** 2020-08-07

**Authors:** Ali R. Ahmadi, Maria Chicco, Marcel van den Berge

**Affiliations:** ^1^Department of Internal Medicine, Erasmus MC, Rotterdam, Netherlands; ^2^Department of Internal Medicine, Admiraal de Ruyter Ziekenhuis, Goes, Netherlands; ^3^Department of General Surgery, Wexham Park Hospital, Frimley Health NHS Trust, Frimley, UK

## Abstract

A male bodybuilder of 39 years of age developed severe pruritus, nausea, and jaundice after injecting anabolic steroids purchased on the black market. The patient had no history of liver disease and no risk factors for viral hepatitis. Extensive laboratory testing, radiographic imaging, and liver biopsy excluded a majority of potential pathologies. The patient was diagnosed with drug-induced acute liver injury and secondary acute renal failure most likely caused by testosterone purchased on the black market. The pruritus caused insomnia and significant psychological distress. Treatment was initiated with cholestyramine and naltrexone for one week with no effect on the pruritus. Subsequently, all medications were stopped, and rifampicin was started. Pruritus resolved after starting rifampicin, and liver and kidney function improved rapidly and normalized within 5 months.

## 1. Background

Drug-induced liver injury (DILI) is the most common cause of acute liver failure in the Western world [1]. It is a rare disease with possible fatal outcome with limited treatment options. Cholestatic pruritus is a common symptom of DILI caused by increased levels of serum bilirubin. It is recognized as a troublesome symptom, which significantly affects patients' daily activities, including sleep and the ability to work or attend school [2]. The negative impact of pruritus on patients' quality of life has been demonstrated in numerous studies: chronic pruritus can lead to anxiety, depression, and even suicide. In this case report, our patient experienced extreme discomfort because of pruritus caused by DILI. He was unable to sleep, which had significant impact on his mood and overall mental status. We used the Roussel Uclaf Causality Assessment Method (RUCAM) to quantitatively assess causality in this suspected case of DILI [3]. RUCAM represents a structured, standardized, validated, and hepatotoxicity-specific diagnostic approach that attributes scores to individual key items, providing final quantitative gradings of causality for each suspect drug/herb in a case report. Based on this scoring system, DILI was likely to be the cause. After several unsuccessful attempts to alleviate pruritus, we resorted to the use of rifampicin with remarkable success. Rifampicin is most commonly used as an antibiotic for treatment of tuberculosis. An additional and not well-known off-label indication of rifampicin is to treat cholestatic pruritus.

## 2. Case Presentation

**Figure 1:**
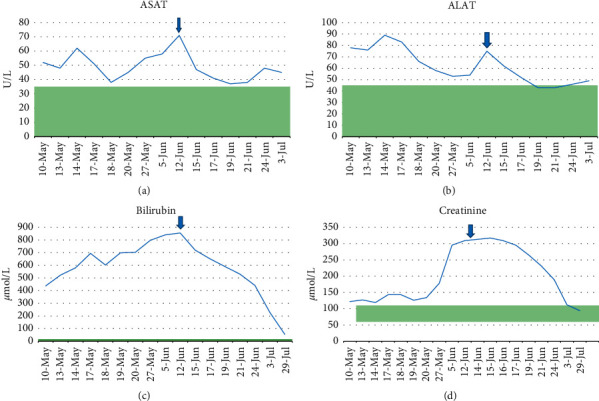
Biochemical parameters. Blue arrow indicates the start of treatment with rifampicin. The green bar indicates the normal range of the different parameters.

The patient saw his general practitioner because of malaise, nausea, and severe generalized pruritus. The patient was referred to the hospital the same day. He had no prior disease history. He had not recently traveled abroad and had no fever or weight loss. The patient mentioned he had loss of appetite, discoloured faeces, and dark “coca-cola-like” urine. He reported use of certain supplements, such as mesterolone, exemestane, and taladafil, which he had acquired from a pharmacy abroad. He also indicated that he had recently injected 250 mg of testosterone enanthate, which he had purchased on the black market. The patient was a bodybuilding enthusiast, who used these drugs to gain muscle mass. However, he had discontinued all medications since the onset of symptoms. On inspection, the patient was noticeably icteric and covered with scratch marks and crusts. On abdominal examination, there was right upper quadrant tenderness but no hepatosplenomegaly or ascites. Laboratory findings were suggestive of acute liver injury and cholestatic disease with significantly deranged AST and ALT. Initially, the patient had normal kidney function; however, his kidney function declined as the serum bilirubin kept increasing. In order to exclude any possibility of autoimmune liver disease, ANA, AMA, SMA, amyloid antibodies, and M-protein analysis were performed which were negative. Serology for hepatitis A, B, C, and E, HIV, CMV, EBV, HSV, and toxoplasmosis was also negative. An abdominal ultrasound showed uncomplicated cholelithiasis with no intra or extrahepatic biliary dilation and a normal liver surface. The kidneys, spleen, and pancreas did not present any abnormalities. In order to exclude obstruction of the biliary tract, a magnetic resonance cholangiopancreaography was also performed which was normal. Eventually, an ultrasound-guided liver biopsy was obtained. The arrows in Figure 1 demonstrate the effect of rifampicin; as soon as rifampicin was introduced, the patient reported substantial improvement in pruritus. His laboratory values (AST, ALT, total bilirubin, and creatinine) also improved.

**Figure 2:**
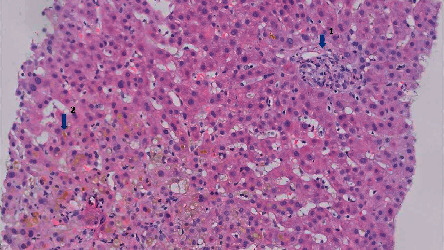
Liver needle biopsy (20X magnification).

Histological analysis of the ultrasound-acquired liver biopsy showed normal liver composition (Figure 2). Lymphocytes, eosinophils, and neutrophils were present in the portal areas indicating acute inflammation (Figure 2, arrow number 1). The images also show cholestasis with swollen periportal hepatocytes and bilirubin retention within hepatocytes (Figure 2, arrow number 2). Furthermore, histology revealed scattered neutrophils and lymphocytes within the liver. These findings correspond with toxin-induced liver injury.

## 3. RUCAM Classification

We first classified the type of liver injury according to the updated RUCAM. By applying the parameters, we concluded that cholestatic liver injury was the most likely cause and proceeded with calculating the total score for the case based on the extensive questionnaire provided in Table 1. A total score of 6 points was established, and this score was associated with DILI being a probable cause of the observed liver injury.

## 4. Differential Diagnosis

During the initial workup, we expected the liver and kidney injuries to be of toxic origin, especially since the patient mentioned that he had injected testosterone purchased on the black market. However, considering the severity of this case, we had to be certain and exclude all other potential causes. In particular, we considered the possibility of biliary obstruction caused by cholelithiasis and viral hepatitis, given the acute onset of symptoms. In order to exclude autoimmune conditions, we performed an autoimmune screen. Because of the rapid decline in liver and renal function, storage diseases such as amyloidosis were excluded. Considering the acute disease onset, patient history and use of illegal testosterone supplements, the absence of any other possible causes, and the results from the liver biopsy, we concluded that this case represented a severe presentation of toxin-induced acute liver injury and secondary acute renal failure most likely caused by bile cast depositions in the kidney. The patient had a severely progressing jaundice, pruritus, and renal failure after removal of the underlying cause, which was considered a rare but typical case of DILI.

## 5. Treatment and Follow-Up

Initially, the patient was given antiemetics and supportive care. Besides that, the patient also received cholestyramine briefly, but this was discontinued because of the unpleasant taste in his mouth and the absence of effect. At a later stage, because of the severe persistent pruritis, the patient was switched to rifampicin twice 300 mg/day per os until near-complete recovery of liver and renal function. Rifampicin was reduced in dosage after the first week to 300 mg/day after the first week and was given for two weeks in total thereafter.

Nearly 5 months after the initial presentation, the patient has completely recovered. The jaundice, pruritus, and nausea have resolved. His laboratory values demonstrate a complete recovery of liver and renal function. Luckily, the patient did not sustain any long-term damage from this severe case of drug-induced acute liver and kidney injury.

## 6. Discussion

Drug-induced liver injury is considered the most common cause of death from acute liver failure (ALF) after paracetamol-induced ALF in the Western world [4]. Due to its variable clinical presentation, it is difficult to identify and is often considered a diagnosis of exclusion. By using the RUCAM methodology, we sought to estimate the likelihood of DILI in this case. Early identification and cessation of the drug causing the liver injury is key. Anabolic steroids represent a major group of drugs that cause liver toxicity. In this case, the suspicion of DILI was high because of the many androgen compounds the patient was taking daily and the testosterone injections twice a week, especially since the testosterone injections were bought on the black market. Similar cases of anabolic steroid liver injury have been described [5].

In general, hepatocellular function resumes within days to weeks after removal of the underlying cause of injury, which can include drugs, toxins, or short-term biliary obstruction. However, in our case, hepatocellular function was not restored. To the contrary, jaundice, pruritus, and kidney function deteriorated leading to DILI with serum bilirubin levels above 800 *μ*mol/L several weeks after the removal of the underlying cause.

Interestingly, as the serum bilirubin increased, the serum creatinine increased as well. The eGFR declined over time from 90 ml/min to 20 ml/min. We suspected that this was caused by bile cast nephropathy. It is a very rare phenomenon with unclear etiology. Bile cast nephropathy is a known secondary dysfunction of the kidneys most likely caused by direct toxicity from bile acids, obstructive physiology from bile casts, and systemic hypoperfusion from vasodilation [6]. Some patients may require dialysis, plasmapheresis, or even kidney transplantation. In our case, the creatinine started decreasing as soon as we initiated rifampicin, and no additional renal replacement therapies were necessary.

In this case, pruritus was the most prevalent symptom reported by the patient. Pruritus is a complex phenomenon, and its exact pathophysiology remains poorly understood. Many factors such as bile acids, histamine, cytokines, immune cells, and receptors are known to play a role. However, effective treatment strategies are lacking [7]. Guidelines on treating pruritus suggest using cholestyramine 4–16 g/day before starting rifampicin as a second line therapy. However, the evidence for using rifampicin is of higher quality as compared to cholestyramine [7, 8]. Several randomized controlled trials have shown the effectiveness of rifampicin against pruritus [9]. Cholestyramine needs to be taken 1 hour prior to a meal and 4 hours prior to any other medication, as it may interfere with their intestinal absorption. It is also known that cholestyramine leaves an unpleasant taste in the mouth. It is often noted that contraindications for rifampicin use are icterus and hepatitis. A comprehensive retrospective review studied the correlation of rifampicin intake and adverse events [10]. In that study, 105 patients had a median rifampicin intake of 131 days with only 5 cases of hepatitis. After stopping rifampicin, the hepatitis resolved spontaneously. Although rifampicin may cause hepatitis in a very small percentage of population, hepatitis is not an absolute contraindication to its use. It is recommended to perform frequent laboratory testing of liver enzymes to detect any adverse events caused by rifampicin. In our case, rifampicin had the opposite effect, whereby liver function and bilirubin clearance instantaneously improved and pruritus resolved.

## 7. Take-Home Messages

- Supplements and, especially, anabolic steroids can cause significant acute liver injury or even liver failure.
- The RUCAM methodology is a useful tool to objectively assess for hepatocellular injury likely caused by DILI.
- Pruritus is an important symptom of cholestatic disease with a significant negative impact on patients' psychological well-being and quality of life.
- Off-label use of rifampicin may benefit patients with cholestatic pruritus, in particular, when other agents are ineffective.
- Care must be taken when using rifampicin, as it is a strong inducer of the enzyme cytochrome P450 and can lead to faster metabolism of other medications. This is especially important with concurrent use of medications that need to maintain adequate serum concentrations.

## Figures and Tables

**Table 1:** Updated RUCAM for the cholestatic or mixed liver injury of DILI and HILI. This table is reproduced from the work of Danan et al. [3] (under the Creative Commons Attribution License/public domain).

Items for cholestatic or mixed liver injury	Score	Result
(1) Time to onset from the beginning of the drug/herb		
(i) 5–90 days (rechallenge: 1–90 days)	+2	**+2**
(ii) <5 or >90 days (rechallenge: >90 days)	+1	□
Alternative: time to onset from cessation of the drug/herb		
(iii) except for slowly metabolized chemicals: ≤30 days	+1	□
(2) Course of ALP after cessation of the drug/herb		
Percentage difference between ALP peak and N		
(i) Decrease ≥50% within 180 days	+2	**+2**
(ii) Decrease <50% within 180 days	+1	□
(iii) No information, persistence, increase, or continued drug/herb use	0	□
(3) Risk factors		
(i) Alcohol use: current drinks/d: >2 for women, >3 for men	+1	□
(ii) Alcohol use: current drinks/d: ≤2 for women, ≤3 for men	0	**0**
(iii) Pregnancy	+1	□
(iv) Age ≥ 55 years	+1	□
(v) Age < 55 years	0	**0**
(4) Concomitant use of drug(s)/herb(s)		
(i) None or no information	0	□
(ii) Concomitant drug/herb with incompatible time to onset	0	□
(iii) Concomitant drug/herb with compatible or suggestive time to onset	−1	**−1**
(iv) Concomitant drug/herb known as hepatotoxin and with compatible or suggestive time to onset	−2	□
(v) Concomitant drug/herb with evidence for its role in this case (positive rechallenge or validated test)	−3	□
(5) Search for alternative causes	Tick if negative	Tick if not done
Group I (7 causes)		
(i) HAV: anti-HAV-IgM	**X**	□
(ii) HBV: HBsAg, anti-HBc-IgM, and HBV-DNA	**X**	□
(iii) HCV: anti-HCV and HCV-RNA	**X**	□
(iv) HEV: anti-HEV-IgM, anti-HEV-IgG, and HEV-RNA	**X**	□
(v) Hepatobiliary sonography/colour Doppler sonography of the liver vessels/endosonography/CT/MRC	**X**	□
(vi) Alcoholism (AST/ALT ≥ 2)	**X**	□
(vii) Acute recent hypotension history (particularly in case of underlying heart disease)	**X**	□
Group II (5 causes)		
(i) Complications of underlying disease(s) such as sepsis, metastatic malignancy, autoimmune hepatitis, chronic hepatitis B or C, primary biliary cholangitis or sclerosing cholangitis, and genetic liver diseases	**X**	□
(ii) Infection suggested by PCR and titer change for		
(iii) CMV (anti-CMV-IgM and anti-CMV-IgG)	**X**	□
(iv) EBV (anti-EBV-IgM and anti-EBV-IgG)	**X**	□
(v) HSV (anti-HSV-IgM and anti-HSV-IgG)	**X**	□
(vi) VZV (anti-VZV-IgM and anti-VZV-IgG)	□	**X**
Evaluation of group I and II		
(i) All causes: groups I and II reasonably ruled out	+2	□
(ii) The 7 causes of group I ruled out	+1	**+1**
(iii) 6 or 5 causes of group I ruled out	0	**0**
(iv) Less than 5 causes of group I ruled out	−2	□
(v) Alternative cause highly probable	−3	□
(6) Previous hepatotoxicity of the drug/herb		
(i) Reaction labelled in the product characteristics	+2	**+2**
(ii) Reaction published but unlabelled	+1	□
(iii) Reaction unknown	0	□
(7) Response to unintentional reexposure		
(i) Doubling of ALP with the drug/herb alone, provided ALP below 2*N* before reexposure	+3	□
(ii) Doubling of ALP with the drugs(s)/herbs(s) already given at the time of first reaction	+1	□
(iii) Increase of ALP but less than *N* in the same conditions as for the first administration	−2	□
(iv) Other situations	0	**0**
Total score for the case		**6**
